# Relationship Between Patient Activation and Type 2 Diabetes Mellitus Self-management and Clinical Outcomes in Saudi Arabian Primary Care Setting

**DOI:** 10.1177/08901171231224889

**Published:** 2023-12-26

**Authors:** Nasser Almutairi, Vinod Gopaldasani, Hassan Hosseinzadeh

**Affiliations:** 1 School of Health & Society, 8691 University of Wollongong, NSW, Australia; 2 Public Health Sector, Ministry of Health, Riyadh, Saudi Arabia

**Keywords:** type 2 diabetes, patient activation, self-management, clinical outcomes

## Abstract

**Purpose:**

Type 2 diabetes mellitus (T2DM) self-management is a comprehensive approach that individuals with T2DM employ to manage and control their condition. Patients’ activation, “an individual’s knowledge, skill, and confidence for managing their health and health care”, has been used as a major driver of self-management. This study aimed to assess the relationship of patient activation with T2DM self-management and clinical outcomes.

**Design:**

A cross-sectional study.

**Subjects:**

Patients with type T2DM who age 18-years and older.

**Setting:**

The primary care centers in Saudi Arabia.

**Measures:**

Patient activation measure (PAM) and the Summary of Diabetes Self-Care Activities (SDSCA).

**Analysis:**

Descriptive statistic, T-test, One-way ANOVA test, Chi-square test, and linear and logistic regressions were performed.

**Results:**

A total of 398 patients, mostly male (54.9%) with a mean age of 53.2 (±10.7) years old participated in the study. The participants’ mean of Hemoglobin A1c (HbA1c) was 8.4% (±1.7%) and most of them (74.5%) had an uncontrolled HbA1c level (>7% %). The mean patient activation score was 55.9 (±13.5). 24.4% were at [PA1], 26.7% at [PA2], 37.4% at [PA3], and 11.5% at [PA4]. Patient activation level was positively associated with better glycemic control and self-management behaviors including diet, physical activity, blood glucose self-testing, foot care, and smoking (*P* < .05) but not with adherence to medication.

**Conclusions:**

Our findings reveal a positive association between patient activation level and enhanced glycemic control and self-management behaviors and suggest that patient activation-informed self-management interventions are more likely to yield promising health outcomes.

## Introduction and Background

Diabetes mellitus is a major global public health concern. In 2021, the International Diabetes Federation (IDF) estimated that there were 537 million adults with diabetes worldwide and it is expected to increase to 783 million by 2045.^1^ The estimated global direct cost of diabetes is US$825 billion per year.^2^ Population growth, aging, urbanization, increasing prevalence of obesity, sedentary lifestyle, and unhealthy diet have been identified as the main factors for the high prevalence of diabetes worldwide.^3^ The most common form of diabetes is type 2 diabetes mellitus (T2DM). It accounts for 90% of all people with diabetes.^4^

Saudi Arabia is witnessing economic growth and technological development that have led to improved health services and optimal control of communicable diseases.^5,6^ However, noncommunicable diseases like diabetes mellitus has started to emerge as a growing public health burden in the country mainly due to rapid changes in lifestyle as well as urbanization during the past 4 decades.^5^ In 2021, IDF estimated the prevalence of diabetes in Saudi Arabia among adults aged 20-79 years was 18.7% which is among the highest prevalence rates in the Middle East and North Africa region. This is nearly twice the global prevalence of diabetes (10.5%) and it is expected to increase to 21.4% by 2045.^1^

Self-management is a comprehensive approach that individuals with diabetes mellitus employ to manage and control their condition. Type 2 diabetes mellitus self-management refers to patient’s capability to manage symptoms, treatment, physical, psychosocial and lifestyle consequences related to T2DM.^7^ It includes activities such as diet, physical activity, blood glucose self-monitoring, foot care and adherence to medication.^8^ Patient activation refers to “an individual’s knowledge, skill, and confidence for managing their health and health care”.^9^ Literature suggested that people who are more activated are more likely to have better self-management behaviors and to practice healthy behaviors such as healthy diet habits, physical activity, and adherence to management plan.^9-14^ Furthermore, a higher level of patient activation is associated with better DM clinical outcomes such as HbA1c (Hemoglobin A1C), HDL (High-Density Lipoproteins), blood pressure, and triglycerides.^10,15,16^ However, to the best of our knowledge, there has been no study that has examined the association between patient activation and T2DM self-management in Saudi Arabia. This study is an attempt to fill this gap by assessing the association of patient activation with T2DM self-management and clinical outcomes in primary care settings in Saudi Arabia.

## Methodology

### Study Design and Setting

This is a cross-sectional study conducted in diabetic clinics at primary care centers in the Alrass City in Saudi Arabia from November 2019 to February 2020. Convenience sampling was utilized to recruit patients attending the primary care diabetic clinics. Sample size was calculated using Cochran’s formula, a widely recognized method for determining an optimal sample size with the desired level of precision.^17^ The parameters considered in the calculation included the anticipated effect size, desired level of confidence, and population size. As a result, the total sample size was estimated to be 398, with a provision for 10% attrition.

### Recruitment and Data Collection

In Saudi Arabia health care system, individuals living with T2DM routinely visit diabetic clinic in primary care centers to check their health status. Patients with T2DM who are attending the clinic were recruited by their doctors to participate in the current study. Participants were recruited from 13 primary care centers around the city. Written consent was taken from the participants. Patients answered a self-administered questionnaire. The inclusion criteria were (a) age ≥18 years old (b) diagnosed with T2DM (c) registered in the primary care center. Participants were excluded if they did not meet the inclusion criteria, were unable to communicate in the Arabic language, or exhibited cognitive impairment as determined through their medical records and clinical evaluations conducted by their doctors.

### Study Measures

• **Demographic data** - age, gender, education, marital status, employment status, and economic status.
• **Clinical data** including HbA1c, blood pressure, lipid profile including cholesterol and triglycerides, body mass index (BMI), type of medication, comorbidities, hospitalization due to T2DM in last 24 months, the duration of T2DM, and any previous diabetes education.
• **Patient activation measure (PAM-13):** Patient Activation Measure (PAM) 13™ is a highly valid and reliable instrument for assessing self-efficacy in patients with chronic disease. Its score ranges between 0 and 100, classifying patients into stage 1 (0 to ≤47), stage 2 (47.1-55.1), stage 3 (55.2 to 67) and stage 4 (≥67.1). Stage 1 is least and Stage 4 is highly activated.^9,18^ Permission to use PAM13 was obtained from Insignia Health.
• **Self-management behaviors**: The Summary of Diabetes Self-Care Activities (SDSCA) measure is a validated multidimensional tool designed to assess adherence to T2DM self-management behaviors.^19^ It has been selected to measure T2DM self-management behaviors for this study. The most recent revised version of SDSCA consists of 11 core items with 14 additional expanded items. For purpose of this study, 13 items were utilized measuring 6 domains: diet (4 items), exercise (2 items), blood sugar testing (2 items), medication adherence (2 items), foot care (2 items), and smoking (1 item). The instrument is self-reported questions about participant’s activities during the last 7 days and based on answering, the score for each domain was calculated in addition to the total score.^20^

### Measures Translation

The SDSCA test have been previously validated and translated to Arabic language with adequate internal consistency and reliability α = .76 and .75.^21,22^ As there was no validated Arabic version of the PAM-13, the measure was translated to Arabic and validated following WHO guidelines of translation and adaptation of instruments.^23^ The process of translation as follow: forward translation, expert panel, backward translation, expert panel, pretesting, and final version. First, the English versions were sent to a translator who is a health professional and has Arabic as his mother tongue language but also knowledgeable about the English terminology used in the instruments. Then, an expert panel, selected by the researcher, reviewed the Arabic versions and made necessary corrections to any inadequate expressions or concepts. The revised Arabic versions were then translated back into English by a native English-speaking translator who had no knowledge of the study, and the back translated versions were found to be mostly similar to the original English versions. After that, an expert panel assessed the content validity, and final versions were prepared for pretesting.

### Statistical Analysis

Data analysis was performed using SPSS version 26. Descriptive statistics were employed to summarize the frequency, distribution, mean, and standard deviation. Chi-square tests assessed associations between categorical variables. T-tests were used to identify significant differences in means between 2 groups, while one-way ANOVA tests were utilized for multiple group comparisons. Linear and logistic regressions analyses were utilized to assess if PAM can predict the health outcome (HbA1c and SDSCA). Statistical significance was determined using a *P-value* <.05.

## Results

### Participants’ Characteristics and Clinical Data

As outlined in Table 1, a total of 398 participants participated in the study. The participants were mostly male (54.9%), married (86.2%), aged between 41-60 years old (62.1%) with a mean of [53.2 ± 10.7] years, from a middle-income family (53.8%) and had a secondary or university education (57.7%). The mean HbA1c was 8.4% ranging from 5.3%-13.4% and approximately three-quarters of the total sample had poor glycemic control, HbA1c > 7% (74.5%). More than half of the participants were obese (53%) and one-third were overweight (33.9%). The mean diabetes duration since diagnosis was 9.7 years, and around 60% of the participants were diagnosed in the past 10 years. With respect to comorbidities, more than one-third had at least one chronic condition beside T2DM (36.3%). The most frequent chronic condition was hypertension (69.8%).

**Table 1.** Participants’ Demographic and Health Outcomes Characteristics (Total Sample Size = 398).

Variable	M (SD)	Category	N (%)
Gender		Male	218 (54.9)
		Female	179 (45.1)
Age	53.2 (10.7)	<= 40	47 (12.6)
		41-60	231 (62.1)
		>60	94 (25.3)
Marital status		Single	15 (3.9)
		Married	332 (86.2)
		Others including divorced and widowed	38 (9.9)
Education		No education	63 (16.7)
		Primary education	97 (25.7)
		Secondary education	87 (23.0)
		University education	131 (34.7)
Employment Status		Employed	144 (37.1)
		Not employed	116 (29.9)
		Retired	128 (33.0)
Family income status^a^		Low income	125 (33.8)
		Middle income	199 (53.8)
		High income	46 (12.4)
Glycemic control (HBA1c)	8.4 (1.7)	Controlled <= 7% Uncontrolled >7%	96 (25.5) 280 (74.5)

Total cholesterol	4.94 (1.12)	Normal <5.18 mmol/L Abnormal >5.18 mmol/L	222 (60.5) 145 (39.5)

Triglyceride	1.75 (.81)	Normal <=1.70 mmol/L Abnormal >1.70 mmol/L	205 (55.7) 16 (44.3)

Diastolic blood pressure	77 (9)	Normal <90 mmHg Abnormal >= 90 mmHg	340 (88.8) 43 (11.2)

Systolic blood pressure	131 (15)	Normal <140 mmHg Abnormal >= 140 mmHg	263 (68.7) 120 (31.3)

Body mass index	30.90 (5.65)	Normal weight 18.5-24.9 Overweight 25-29.9 Obese >=30	51 (13.2) 131 (33.9) 205 (53)

	
Diabetes duration	9.7 (7.2)	<5 years 5-10 years >10 years	118 (31.4) 105 (27.9) 153 (40.7)

	
Type of medication		Insulin only Oral hypoglycemic agent only Insulin+oral hypoglycemic agent	26 (6.6) 268 (67.7) 102 (25.8)

	
Hospitalization du to DM		Yes No	40 (10.5) 340 (89.5)
Previous health education		Yes No	47 (11.8) 351 (88.2)
Comorbidities		Yes	136 (36.3)
		No	239 (63.7)
Smoking		Yes	33 (8.5)
		No	354 (91.5)

^a^Family income is per month and classified into 3 groups: low income (<5000 SAR); middle income (10 000-15 000 SAR) and high income (>15 000 SAR). 1 SAR = .35 AUD. Saudi family income data (https://www.stats.gov.sa/sites/default/files/household_income_and_expenditure_survey_2018_en_27-6-2019.pdf).

## Participants’ Activation Levels

As presented in Table 2, the mean PAM score was [55.9 ± 13.5] ranged from (14.5-100) and 24.4% of the participants were at [PAM 1], 26.7% at [PAM 2], 37.4% at [PAM 3], and 11.5% at [PAM 4].

**Table 2.** Patient Activation Distribution of Responses, Mean and Standard Deviation.

PAM13	Strongly Disagree N (%)	Disagree N (%)	Agree N (%)	Strongly Agree N (%)	Not Applicable N (%)
1. When all is said and done, I am the person who is responsible for taking care of my health	4 (1.0)	11 (2.8)	257 (65.7)	116 (29.7)	3 (.8)
2. Taking an active role in my own health care is the most important thing that affects my health	9 (2.3)	29 (7.4)	241 (61.5)	100 (25.5)	13 (3.3)
3. I am confident I can help prevent or reduce problems associated with my health	5 (1.3)	49 (12.5)	255 (64.9)	79 (20.1)	5 (1.3)
4. I know what each of my prescribed medications do	7 (1.8)	35 (8.9)	240 (61.1)	106 (27.0)	5 (1.3)
5. I am confident that I can tell whether I need to go to the doctor or whether I can take care of a health problem myself	9 (2.3)	59 (15.0)	242 (61.6)	70 (17.8)	13 (3.3)
6. I am confident that I can tell a doctor or nurse concerns I have even when he or she does not ask	3 (.8)	34 (8.7)	251 (64.0)	100 (25.5)	4 (1.0)
7. I am confident that I can carry out medical treatments I may need to do at home	9 (2.3)	66 (16.8)	237 (60.3)	70 (17.8)	11 (2.8)
8. I understand my health problems and what causes them	4 (1.0)	53 (13.5)	251 (63.9)	72 (18.3)	13 (3.3)
9. I know what treatments are available for my health problems	13 (3.3)	88 (22.4)	223 (56.9)	50 (12.8)	18 (4.6)
10. I have been able to maintain lifestyle changes, like healthy eating or exercising	12 (3.1)	69 (17.6)	226 (57.7)	82 (20.9)	3 (.8)
11. I know how to prevent problems with my health	7 (1.8)	83 (21.1)	236 (60.1)	50 (12.7)	17 (4.3)
12. I am confident I can work out solutions when new problems arise with my health	10 (2.5)	114 (29.0)	219 (55.7)	39 (9.9)	11 (2.8)
13. I am confident that I can maintain lifestyle changes, like healthy eating and exercising, even during times of stress	34 (8.7)	94 (23.9)	202 (51.4)	59 (15.0)	4 (1.0)

PAM level	n (%)
1	96 (24.4)
2	147 (26.7)
3	105 (37.4)
4	45 (11.5)
Total PAM score	*M = 55.9, SD= (13.5)*

*Abbreviations*: M = Mean, SD = Standard Deviation.

### Self-management Behaviors

The means scores of general diet and specific diet subscales were 3.20 ((out of 7) ± 2.30) and 3.46 ((out of 7) ± 1.51) respectively. The overall diet score was 3.33 ((out of 7) ± 1.6). The lowest scores ascribed to blood glucose self-testing 2.63 ((out of 7) ± 2.25) and exercise 2.80 ((out of 7) ± 2.3), whereas the highest score was ascribed to adherence to medication 6.61 ((out of 7) ± 1.25). With respect to foot care, the mean score was 3.15 ((out of 7) ± 2.64). Only 8.5% were smokers with an average cigarette of 16 ± 8/week. The overall SDSCA score was 3.65 ((out of 7) ± 1.2) (Table 3).

**Table 3.** Descriptive Statistics and the Frequency of the Summary of Diabetes Self-Care Activities (SDSCA) Test, (Total Sample Size = 398).

Question	Score Frequency									Total Score M SD
	Days	0	1	2	3	4	5	6	7	
General diet										3.20 (2.30)
Daily healthy diet/past week	n (%)	87 (22.3)	34(8.7)	45(11.5)	53 (13.6)	42(10.7)	50(12.8)	25(6.4)	55(14.1)	
Average daily healthy diet/month	n (%)	72 (18.3)	40(10.2)	45(11.5)	53 (13.5)	55(14.0)	49(12.5)	28(7.1)	51(13.0)	
Specific diet										
Daily fruits and vegetables/past week	n (%)	74 (18.8)	54(13.7)	65(16.5)	59 (15.0)	41(10.4)	26(6.6)	25(6.3)	50(12.7)	3.46 (1.51)
Daily fat meat/past week	n (%)	27 (6.9)	64(16.3)	97(24.7)	76(19.3)	38(9.7)	37(9.4)	13(3.3)	41(10.4)	
Overall diet score										3.33 (1.6)
Exercise										2.80 (2.31)
Daily routine exercise/past week	n (%)	95 (24.2)	44(11.2)	48(12.2)	40 (10.2)	51(13.0)	36(9.2)	16(4.1)	63(16.0)	
Daily structured exercise/past week	n (%)	133 (33.9)	34(8.7)	49(12.5)	44 (11.2)	34(8.7)	28(7.1)	16(4.1)	54(13.8)	
Blood glucose self-testing										2.63 (2.25)
Daily blood glucose testing/past week	n (%)	81 (20.7)	63(16.1)	56(14.3)	66 (16.8)	33(8.4)	24(6.1)	8(2.0)	61(15.6)	
Blood glucose testing recommended by doctor/past week	n (%)	112 (28.6)	60(15.3)	48(12.3)	61 (15.6)	35(9.0)	16(4.1)	10(2.6)	49(12.5)	
Foot care										3.15 (2.64)
Daily feet inspection/past week	n (%)	123 (31.5)	39(10.0)	34(8.7)	34 (8.7)	20(5.1)	11(2.8)	7(1.8)	123(31.5)	
Daily shoes inspection/past week	n (%)	131 (33.6)	40(10.3)	30(7.7)	27 (6.9)	19(4.9)	16(4.1)	7(1.8)	120(30.8)	
Medications										6.61 (1.25)
Insulin taking/past week	n (%)	2 (1.6)	0(.0)	0(.0)	2 (1.6)	0(.0)	2(1.6)	6(4.8)	113(90.4)	
Hypoglycemic tablets/past week	n (%)	9 (2.5)	1(.3)	1(.3)	5 (1.4)	4(1.1)	14(3.8)	12(3.3)	320(87.4)	
Overall score										3.65 (1.2)
Smoking										
Smoking/past week	Yes	33 (8.5%)			Number of cigarettes					16 (8)
	No	354 (91.5%)								

*Abbreviations*: M = Mean, SD = Standard Deviation.

### Associations of PAM with Demographics and Clinical Outcomes

For the purpose of this analysis, PAM levels were recategorized into 2 groups: low activation (PAM 1 and 2) and high activation (PAM 3 and 4). Only education was significantly associated with PAM levels *P* = .001. Participants with higher education were more likely to have high PAM level. There was no significant association between PAM and other demographics including gender, age, employment and marital status, and family income.

With regard to clinical outcomes, only glycemic control and previous health education were significantly associated with PAM levels *P* < .05. Participants with glycemic control or received previous health education were more likely to have a high level of PAM. There was no significant association between PAM and other clinical outcomes including BMI, systolic/diastolic blood pressure, total cholesterol, triglyceride, diabetes duration, type of medication, hospitalization due to diabetes, and presence of comorbidities (Table 4).

**Table 4.** Patient Activation Levels and Glycemic Control Across Different Participants Characteristics (Total Ample Size = 398).

	PAM 1-2 Low Activation		PAM 3-4 High Activation		*P* Value	Glycemic Well-Controlled *HbA1c* <= 7%		Glycemic Un-controlled *HbA1c* > 7%		*P* Value
	N	(%)	N	(%)		N	(%)	N	(%)	
Gender										
Male	126	(58.3)	90	(41.7)	.10	61	(28.9)	150	(71.1)	.096
Female	117	(66.5)	59	(33.5)		35	(21.3)	129	(78.7)	
Age										
<= 40	30	(63.8)	17	(36.2)	.87	9	(22.5)	31	(77.5)	.84
41-60	141	(62.1)	86	(37.9)		59	(26.5)	164	(73.5)	
>60	56	(59.6)	38	(40.4)		22	(24.4)	68	(75.6)	
Employment status										
Employed	81	(56.3)	63	(43.8)	.06	36	(25.7)	104	(74.3)	.31
Not employed	79	(70.5)	33	(29.5)		22	(21.2)	82	(78.8)	
Retired	75	(59.1)	52	(40.9)		37	(30.1)	86	(69.9)	
Education										
No education	50	(80.6)	12	(19.4)	.001	10	(17.9)	46	(82.1)	.006
Primary education	58	(61.7)	36	(38.3)		15	(16.1)	78	(83.9)	
Secondary education	53	(60.9)	34	(39.1)		22	(26.8)	60	(73.2)	
Higher education	67	(51.1)	64	(48.9)		45	(35.2)	83	(64.8)	
Family income										
Low income	80	(65.6)	42	(34.4)	.11	16	(13.9)	99	(86.1)	.002
Middle income	118	(59.3)	81	(40.7)		57	(30.0)	133	(70.0)	
High income	22	(47.8)	24	(52.2)		16	(35.6)	29	(64.4)	
Marital status										
Single	7	(53.8)	6	(46.2)	.80	1	(7.7)	12	(92.3)	.23
Married	202	(61.4)	127	(38.6)		87	(27.3)	232	(72.7)	
Others	22	(57.9)	16	(42.1)		7	(21.2)	26	(78.8)	
Glycemic control										
uncontrolled	180	(65.0)	97	(35.0)	.02		--	--		--
well-controlled	48	(51.1)	46	(48.9)			--	--		--
Body mass index										
Normal weight	26	(52.0)	24	(48.0)	.31	19	(37.3)	32	(62.7)	.04
Overweight	84	(64.1)	47	(35.9)		32	(25.8)	92	(74.2)	
Obese	125	(62.2)	76	(37.8)		43	(22.1)	152	(77.9)	
Systolic BP										
Abnormal	77	(65.3)	41	(34.7)	.51	65	(25.6)	189	(74.4)	.84
Normal	161	(61.7)	100	(38.3)		30	(26.5)	83	(73.5)	
Diastolic BP										
Abnormal	26	(61.9)	16	(38.1)	.90	84	(25.8)	241	(74.2)	.96
Normal	212	(62.9)	125	(37.1)		11	(26.2)	31	(73.8)	
Total cholesterol										
Abnormal	88	(62.0)	54	(38.0)	.95	69	(31.2)	152	(68.8)	.003
Normal	137	(62.3)	83	(37.7)		25	(17.5)	118	(82.5)	
Triglyceride										
Abnormal	99	(61.9)	61	(38.1)	.97	65	(32.2)	137	(67.8)	.002
Normal	126	(62.1)	77	(37.9)		29	(18.1)	131	(81.9)	
Diabetes duration										
<5 years	64	(54.7)	53	(45.3)	.12	41	(36.6)	71	(63.4)	.005
5 - 10 years	62	(60.2)	41	(39.8)		20	(20.2)	79	(79.8)	
>10 years	101	(66.9)	50	(33.1)		31	(20.8)	118	(79.2)	
Type of medication										
Insulin only	15	(65.2)	8	(34.8)	.31	4	(16.0)	21	(84.0)	<.001
Oral hypoglycemic agent only	158	(59.2)	109	(40.8)		82	(32.4)	171	(67.6)	
Insulin+oral hypoglycemic agent	69	(67.6)	33	(32.4)		10	(10.4)	86	(89.6)	
Hospitalization du to diabetes										
Yes	23	(59.0)	16	(41.0)	.68	2	(5.3)	36	(94.7)	.003
No	210	(62.3)	127	(37.7)		87	(27.0)	235	(73.0)	
Previous health education										
Yes	16	(34.0)	31	(66.0)	<.001	13	(28.3)	33	(71.7)	.65
No	227	(65.6)	119	(34.4)		83	(25.2)	247	(74.8)	
Comorbidities										
Yes	148	(62.4)	89	(37.6)	.20	56	(24.2)	175	(75.8)	.22
No	74	(55.6)	59	(44.4)		38	(30.2)	88	(69.8)	

Binary logistic regressions were performed to assess whether patient activation level (with and without covariates) can predict glycemic control. Model 1 (without covariates) was statistically significant (x^*2*^) = 5.65, *P = .01)*. Patient activation level significantly explained variation in glycemic control (*P* = .02). High activated participants were 1.78 times more likely to have a normal glycemic control 95% CI [1.11-2.86]. When covariates were included, the model remained statistically significant (*x*^*2*^ *= 23.17, P = .02).* The predicting power of patient activation level improved (*P* = .02) however, none of the socio-demographic variables were significant. Patient activation level was not a significant predictor of diastolic/systolic blood pressure, cholesterol, triglycerides, and BMI. The result remained consistent with and without inclusion of covariates (*P* > .05).

### Associations of SDSCA with Demographics and Clinical Outcomes

The analysis revealed that male participants had significantly higher diet score (M = 3.5, SD = 2.2) and physical activity score (M = 3.1, SD = 2.3) compared to their female counterparts (M = 2.9, SD = 2.3) and (M = 2.4, SD = 2.3), respectively. Additionally, the researchers found that participants with university education (M = 3.7, SD = 2.1), high family income (M = 3.9, SD = 2.0) and married (M = 3.3, SD = 2.3) were more likely to have a better diet score (*P* < .05).

Clinically, the researchers found that participants who received a previous health education were more likely to have a greater score in general diet (M = 4.1, SD = 1.6) and blood glucose self-testing (M = 3.9, SD = 2.4). Also, participants with well-controlled blood glucose (M = 3.7, SD = 1.6) had a greater diet score compared to those with uncontrolled blood glucose (M = 3.2, SD = 1.6) (*P = .01*). Participants with normal weight had greater diet score (M = 3.8, SD = 1.5) and physical activity score (M = 3.5, SD = 2.4) than those with abnormal weight (*P < .05).*

### Comparison of SDSCA Scores Across PAM Groups

T-test was conducted to detect significant differences in the mean SDSCA scores between 2 PAM groups (low and high activation). As indicated in Table 5, significant differences were observed between the 2 PAM groups in the mean SDSCA overall scores and subscales, which included general diet, specific diet, overall diet, physical activity, blood glucose self-testing, and foot care (*P* < .05). However, no significant difference was found in adherence to medication between the 2 PAM groups (*P* = .6). Also, smoking was found to be associated with low level of activation.

**Table 5.** T Test and Chi-Square Test for the Association Between Diabetes Self-Management and PAM.

	PAM Low Activation		PAM High Activation		t test, *P* Value
	M	SD	M	SD	
General diet	2.75	2.23	3.95	2.22	*t* (387) = 5.17, *P < .001*
Specific diet	3.22	1.45	3.86	1.51	*t* (389) = 4.15, *P < .001*
Physical activity	2.46	2.24	3.35	2.33	*t* (388) = 3.76, *P < .001*
Blood glucose self-testing	2.42	2.11	3	2.45	*t* (387) = 2.64, *P* = *.04*
Foot care	2.90	2.59	2.89	2.59	*t* (386) = 2.61, *P* = *.009*
Medication adherence	6.59	1.26	6.65	1.25	*t* (368) = .4, *P = .64*
Overall score	3.40	1.17	4.07	1.13	*t* (380) = 5.48, *P < .001*
Smokers	n	%	n	%	x^2^ (1, N = 385) = 3.93, *P = .04*
	25	10.5	7	4.8	

PAM low activation: patient activation level 1 and 2.

PAM high activation: patient activation level 3 and 4.

*Abbreviations*: M = Mean, SD = Standard deviation.

Simple and multiple linear regression were performed to assess if patient activation level (with and without covariates) can predict the likelihood that participants have better self-management behaviors. Patient activation level was significantly associated with better general diet (*β* = .25, *P* < .001), specific diet (*β* = .21, *P* < .001), exercise (*β* = .19, *P* < .001), blood glucose self-testing (*β* = .12, *P* < .001) and foot care (*β* = .13, *P* < .001). Patient activation level accounted for 6% of the variance (*R*^*2*^) in general diet score, 4% of the variance in specific diet score and 2% of the variance in blood glucose self-testing and foot care. After all covariates were included in multiple linear regression, the result remained consistent.

## Discussion

### Patient Activation Levels

This study aimed to assess the association of patient activation with T2DM self-management and clinical outcomes in primary care settings in Saudi Arabia. The researchers found that the mean PAM score was 55.9 and 24.4% was at level 1, 26.7% at level 2, 37.4% at level 3, and 11.5% at level 4. These results of PAM level distribution are consistent with those of other studies assessing PAM in people with chronic conditions in China,^24^ Korea,^25^ and US.^14^ These consistent results across diverse populations suggest a certain universality in the patterns of patient activation among individuals with chronic conditions. However, it’s essential to consider the contextual factors that may influence these levels and the implications for tailoring interventions to the Saudi Arabian population.

In terms of the association of PAM with demographic factors, in agreement with other studies,^26,27^ the researchers found that PAM was significantly associated with the level of education. People with better education are more likely to be more activated. In contrast to earlier findings that suggested that younger people are more activated than older people,^18,28^ no significant association between PAM and age was detected. This finding may be attributed to the unique sociocultural factors within our study population. It underscores the multifaceted nature of patient activation and highlights the need for a more understanding of how demographic variables, including education and age, interact in our specific context.

Regarding the association between PAM and gender, number of studies presented mixed results.^29,30^ Our study indicated no statistical significant association between patient activation and gender which is similar to the findings of the study by Hendriks et al targeting type 2 diabetes participants.^31^

With regard to the association between PAM and clinical outcomes, only glycemic control was significantly found to be associated with PAM. People with good glycemic control tend to be more activated. A similar finding was identified by Greene and Hibbard,^10^ Rogvi et al,^32^ Remmers et al^33^ and Milo et al.^34^ Regression analysis indicated that PAM can significantly predict glycemic control among patients. A possible explanation for this finding is that activated patients are more likely to engage in healthy behaviors which might result in better clinical outcomes.^12^ Furthermore, the researchers found that people who previously received structured health education were more likely to be at a high level of activation, although the details of provided health education were unspecified.

Our findings suggest that other clinical outcomes including BMI, systolic/diastolic blood pressure, total cholesterol, triglyceride, diabetes duration, type of medication, hospitalization due to diabetes, and comorbidities were not significantly associated with PAM. These results differ from some previous published studies.^10,15,16,33^ It’s important to note that a considerable portion of our study population exhibited readings of clinical outcomes within the normal range which may partially explain the lack of significant associations in our findings. However, Further research in our Saudi Arabian setting, considering the influence of patient activation on clinical outcomes, is warranted to gain a more comprehensive understanding of the dynamics between patient activation and clinical outcomes.

### Self-management Behaviors

Our findings suggested that the majority of patients with T2DM have relatively low levels of self-management behaviors. Only 12.6% of the participant stated adhering to a recommended diet 7 days/week. A similar result was seen in exercise (14.9%) and blood glucose self-testing (14%). Interestingly, 88.9% of the participants reported adhering to medication 7 days/week. Similar findings were observed in a number of studies in Saudi Arabia from different cities and settings.^35-39^ In these studies, the overall scores of self-management behaviors ranged from (3.5-3.7). In line with our findings, the highest self-management behavior score in these studies was adherence to medication ranging from (4.2-6.8), while exercise was among the lowest along with blood glucose self-testing. This indicates that patients with T2DM completely rely only on medication in managing their diseases and alternatively, they neglect the importance of exercise, blood glucose self-testing, and diet. In addition to adherence to medication, foot care was the most frequently performed self-management behavior. This can be explained by the Islamic practice of ablution in which individuals washes parts of their body including feet 5 times a day before the prayer.^38^

In terms of the association between self-management behaviors and demographic data, gender seemed to be a significant factor affecting self-management behaviors. Males were generally better in self-management behaviors than females. However, the difference was statistically significant on general diet and physical activity behaviors only. However, previous studies suggested that females were more likely to engage with healthy diet, foot care, blood glucose self-testing,^39^ disease prevention behaviors, health-promoting behaviors, symptom recognition, and exercise compared to their male counterparts.^40,41^ The observed inconsistencies, therefore, emphasize the need for further research in diverse contexts to better understand the complexities of gender-related disparities in self-management behaviors.

Furthermore, education level was found to be significantly associated with better self-management behaviors particularly for diet and physical activity. Well-educated people were more likely to practice a healthy diet and regular physical activity. This can be explained by the fact that more education may assist individuals in understanding the importance of diet and physical activity in managing diabetes.^42^ In addition, people with a high level of education generally fall into a higher level of income allowing them better access to healthcare services and resources. Similar findings were reported by Aschalew et al^43^ who found that the odds of poor self-management behaviors for participants with no education and those with a primary level of education were 3.36 and 2.62 times higher than those with a secondary level of education and higher, respectively. Another important finding was that family income was associated with the diet subscale only in which people with low family income were less likely to be engaged in a healthy diet plan. This rather might be attributed to a lack of social support and access to healthy foods.^44^

Clinically, the researchers found that participants with well-controlled glycemia had a greater diet score compared to those with uncontrolled glycemia. However, there was no significant association between glycemic control and other self-management behaviors including physical activity, blood glucose self-testing, foot care, and adherence to medication. A study conducted in Saudi Arabia indicated that those who had good glycemic control were more likely to adhere to a healthy diet and monitor their blood glucose more frequently. Similar to our findings, the study also indicated that adherence to medication, physical activity, and foot care had no significant association with glycemic control.^39^

In line with previous findings by Khaliq et al,^45^ our finding suggests that participants with normal weight were more likely to follow recommended healthy diet and practice regular physical activity. This can be explained by that individuals who consumes healthy foods and are physically active will maintain their normal body weight.^46^

### Associations of Self-management Behaviors with PAM

With regard to the association between self-management behaviors and PAM, the researchers found that participants who followed a healthy diet, were physically active, not smokers, perform regular blood glucose self-testing, and taking care of their foot were more likely to be in a high level of PAM. These findings are supported by the previous studies’ results. Mosen et al^14^ reported that, in adults with chronic conditions, individuals with high PAM scores were significantly more likely to perform self-management behaviors and report high medication adherence than individuals with the lowest PAM scores. Furthermore, Hibbard and Tusler^47^ suggested that PAM can predict change in diabetes self-management behaviors particularly, adherence to healthy diet and medications, exercise, blood glucose self-testing, foot care and stress management. However, our findings suggested that there is no significant association between adherence to medication and PAM, this can be explained by strong ceiling effects as the vast majority of the participants reported a high score of medication adherence [mean = 6.6].^48^

## Strength and Limitation

Our study has several strengths including being the first study, to our knowledge, assessing patient activation in patients with T2DM in Saudi Arabia. This may open the door for future studies with different designs and targeting other chronic conditions. Furthermore, it’s worth noting that the participants in this study were thoughtfully recruited from 13 primary care centers spanning various areas. This approach was deliberate, designed to ensure diversity in demographic, clinical, and cultural variables. As a result, our study findings exhibit potential for generalizability to the wider Saudi population. However, future studies replicating this research in other regions are recommended to foster a more profound understanding of these associations within varying contexts. A potential limitation of this study is being cross-sectional in design hence causal relationship between variables was not determined so future longitudinal or experimental studies are required.

## Conclusion

The current study assessed the association between patient activation and T2DM self-management behaviors and clinical outcomes. The results indicated that high activation level was significantly associated with better T2DM self-management behaviors including (diet, physical activity, blood glucose self-monitoring and foot care), and better glycemic control. These findings offer preliminary insights with potential implications for health promotion practice. They suggest that tailoring interventions based on patient activation levels may yield promising outcomes.
